# A novel GNAS mutation in pseudohypoparathyroidism type 1a with articular flexion deformity: A case report

**DOI:** 10.1515/biol-2022-0918

**Published:** 2024-07-24

**Authors:** Jinxing Wan, Dongjuan He, Jun Xie, Zhizhi Chen

**Affiliations:** Department of Endocrinology, The Quzhou Affiliated Hospital of Wenzhou Medical University, Quzhou People’s Hospital, Quzhou, 324000, Zhejiang, China; Department of Orthopedics, The Quzhou Affiliated Hospital of Wenzhou Medical University, Quzhou People’s Hospital, No. 100 of Minjiang Avenue, Quzhou, 324000, Zhejiang, China; Department of Neurology, The Quzhou Affliliated Hospital of Wenzhou Medical University, Quzhou People’s Hospital, Quzhou, 324000, Zhejiang, China

**Keywords:** POH overlap syndrome, congenital hypothyroidism, inheritance GNAS mutation, pseudohypoparathyroidism, Albright hereditary osteodystrophy, deformity

## Abstract

Pseudohypoparathyroidism (PHP) type 1a (PHP 1a) is a rare hereditary disorder characterized by target organ resistance to hormonal signaling and the Albright hereditary osteodystrophy (AHO) phenotype, which features round facial features, short fingers, subcutaneous calcifications, short stature, obesity, and intellectual disability. Progressive osseous heteroplasia (POH) is another rare disorder characterized by heterotopic ossification (HO) that progressively affects skin, subcutaneous tissues, and deep skeletal muscle. PHP 1a is inherited maternally due to a GNAS mutation, while pure POH is inherited paternally. This case study presented a Chinese boy with congenital hypothyroidism, tonic-clonic seizures, hypoparathyroidism, AHO, POH, and joint fixation deformity. Sequencing analysis of GNAS-Gsα revealed a heterozygous C.432+2T>C(P.?) variant (NM_000516.7) affecting the canonical splice donor site of intron 5 in the boy and his mother, indicating maternal inheritance of a GNAS mutation. The patient was diagnosed with POH overlap syndrome (POH/PHP 1a). Following calcium and calcitriol supplementation, he experienced a reduction in seizures, and surgery was performed to correct the joint fixation deformity caused by HO. This case report provided valuable insights into the genotype-phenotype correlations of POH overlap syndrome and underscored the significance of genetic testing in diagnosing rare diseases.

## 1 Introduction

**Figure 1:**
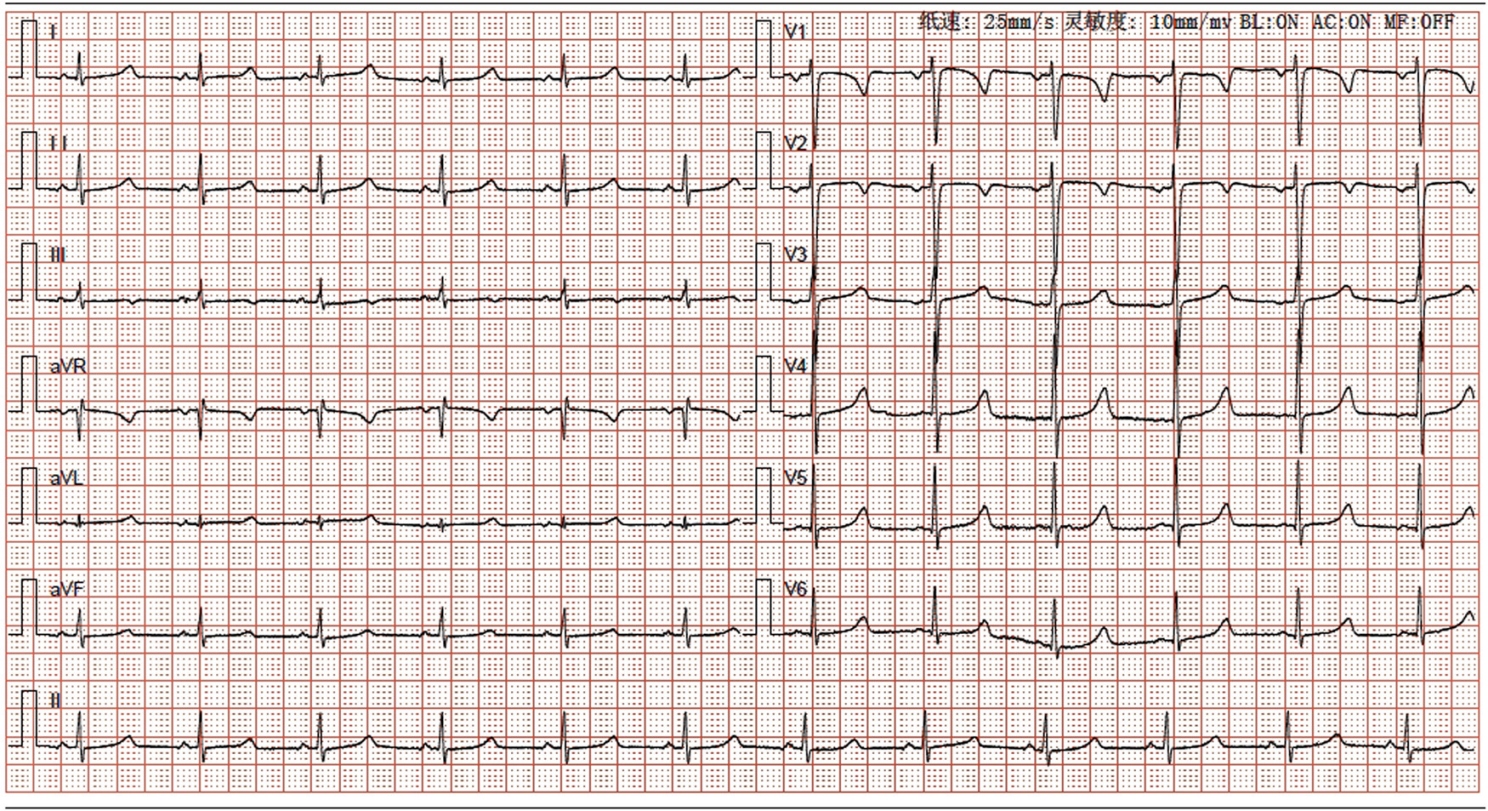
ECG result of the patient. The sensitivity of the ECG machine was 10 mm/mV. The speed of the report paper was 25 mm/s.

**Figure 2:**
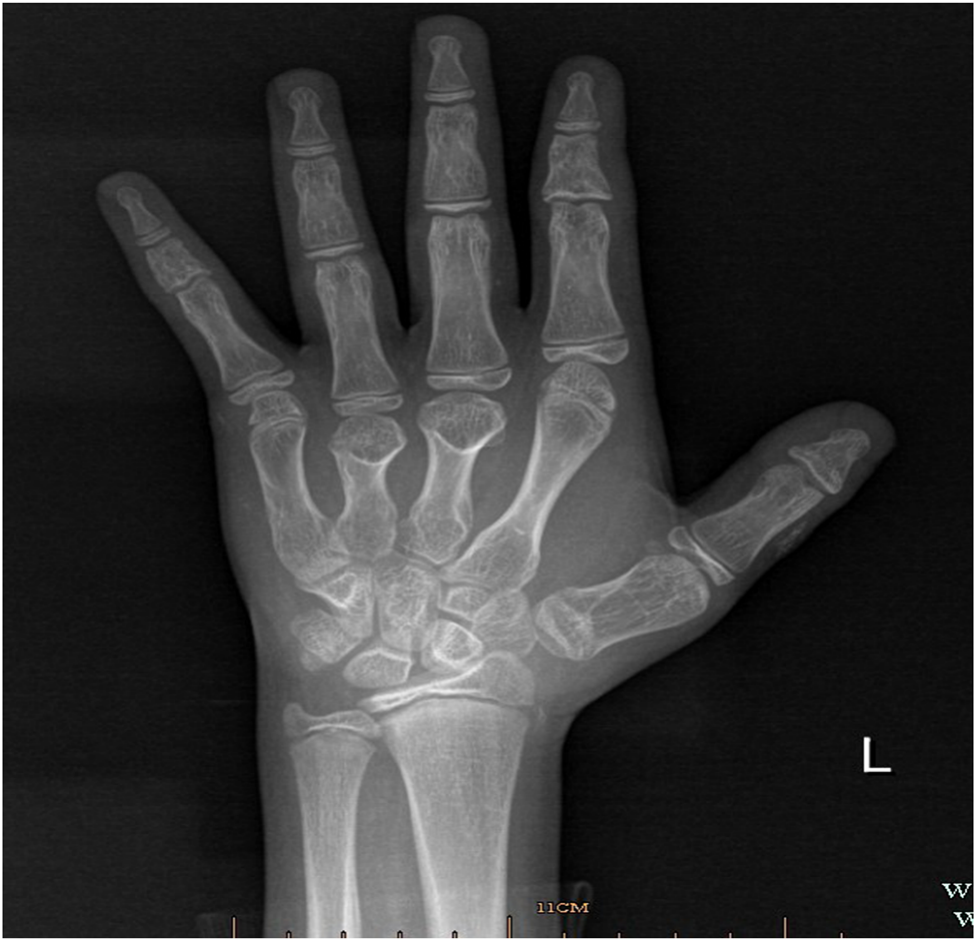
The left-hand X-ray result of the patient.

**Figure 3:**
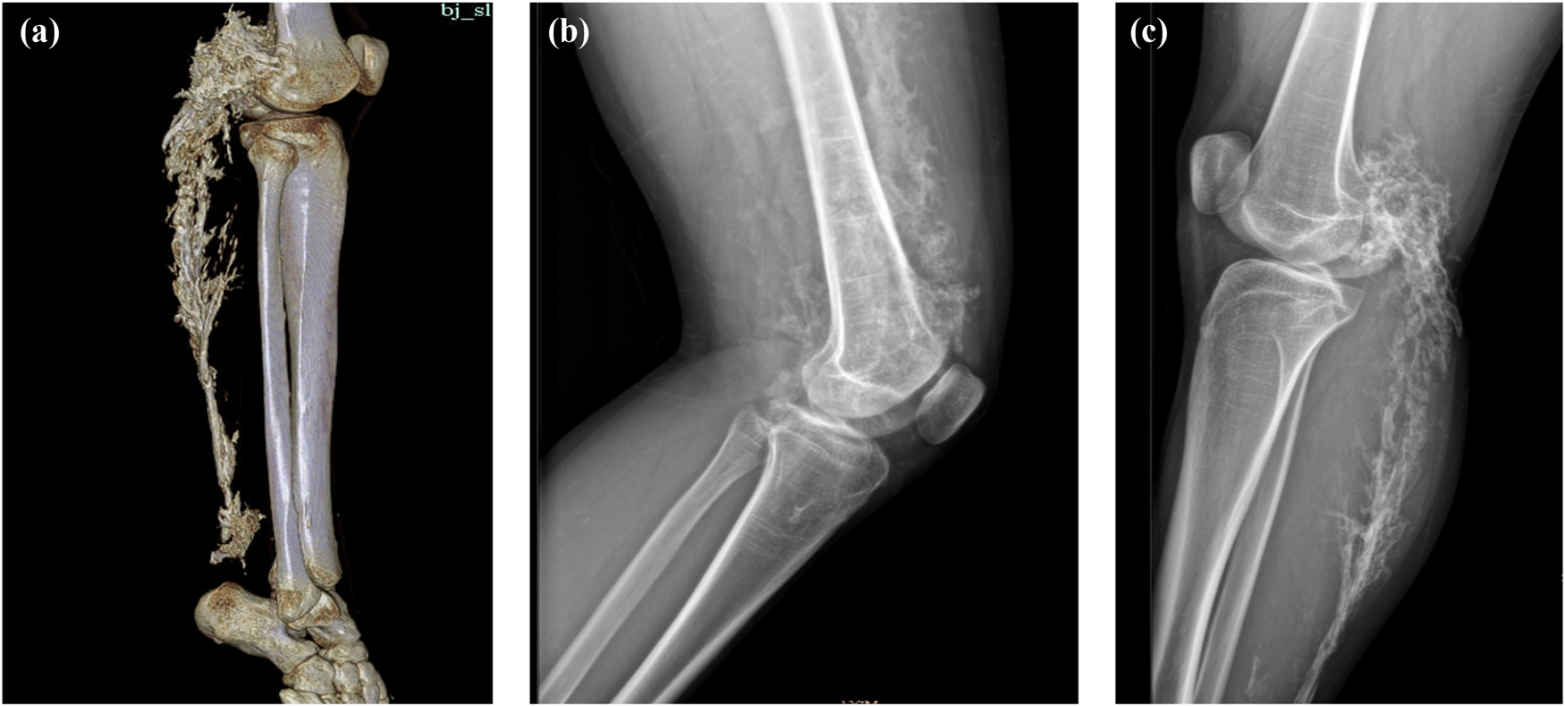
The three-dimensional reconstruction image (a) and the CT image (b and c) of the right knee joint of the patient showed multiple calcifications.

**Figure 4:**
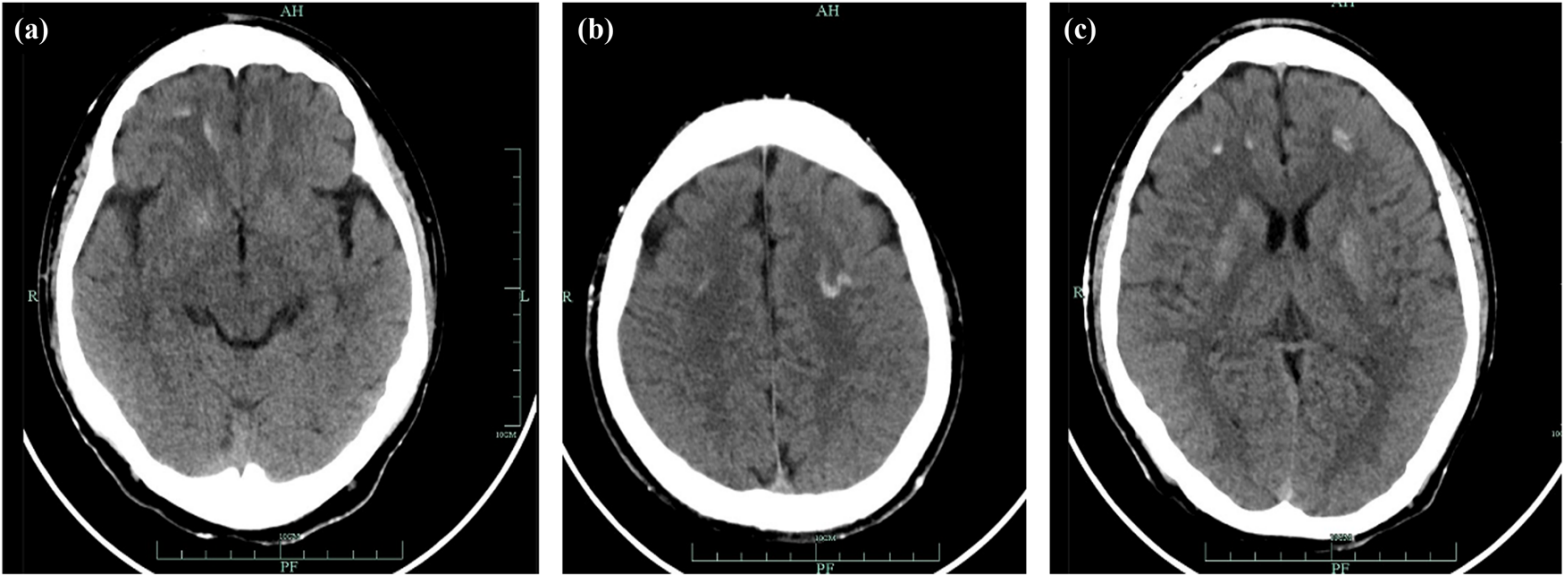
The head CT scan image (a–c) of the patient showed multiple calcifications.

Pseudohypoparathyroidism (PHP) and related disorders, collectively known as inactivating parathyroid hormone (PTH)-related peptide (PTH/PTHrP) signaling disorders (iPPSD), are rare conditions caused by genetic or epigenetic defects at the GNAS complex locus on chromosome 20q13.3, which impair the function of the stimulatory G protein (Gsα) or lead to abnormal Gsα expression and other splice variants [1]. The global prevalence of PHP remains unclear, but it is estimated to be 1.2/100,000 in Japan, 1/150,000 in Italy, 1.1/100,000 in Denmark, and 1/20,000 in the United States [2,3]. Genetic testing can help exclude other potential illnesses when diagnosing PHP. Albright hereditary osteodystrophy (AHO), a disorder caused by GNAS inactivation, is characterized by a round face, small digits, short stature, subcutaneous calcifications, and mental impairment. Progressive osseous heteroplasia (POH) is characterized by progressive heterotopic ossifications (HOs) of the dermis, skeletal muscle, and deep connective tissues [4]. Fixed flexion deformity is a rare juvenile deformity that is seldom documented in PHP cases as a deformity resulting from subcutaneous calcification. It is uncommon to encounter generalized calcinosis in children that leads to a fixed flexion deformity. This case study presented a rare case of a boy with clinical features of POH overlap syndrome (POH/PHP 1a) and a GNAS-inactivating mutation. The case underscored the significance of genetic testing in diagnosing rare diseases such as this and provided insights into the clinical characteristics of POH and PHP 1a (Figures 1–4).

### 1.1 Case report

**Figure 5:**
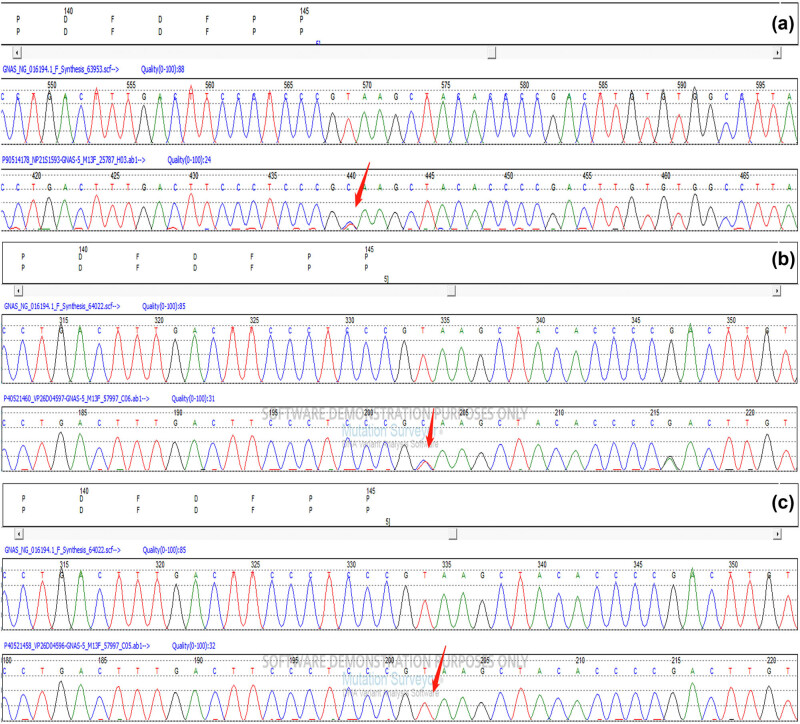
(a) Genetic test result of the patient showed splicing mutation GNAS C.432+2T>CP.?. The red arrow points to the mutation site. (b) Genetic test result of the patient’s mother. (c) Genetic test result of the patient’s father.

**Figure 6:**
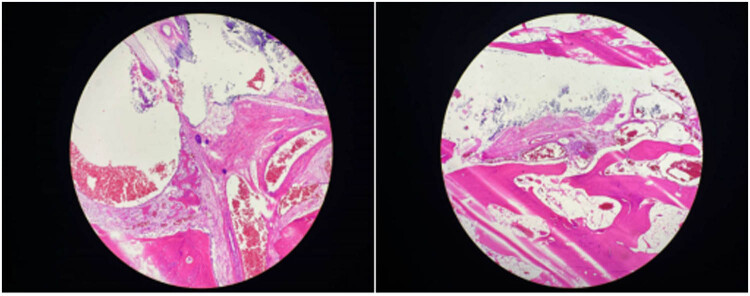
Postoperative pathology images of the soft tissues and fibroadipose tissues in lower leg of the patient.


The 12-year-old male patient in this case had a history of congenital hypothyroidism and was treated with levothyroxine tablets from birth. Despite normal academic achievement, the patient’s growth and development were delayed compared to his peers. At 12 years and 5 months of age, he experienced a generalized tonic-clonic seizure and was admitted to the hospital. His parents were not consanguineous in marriage. Physical examination revealed several clinical characteristics of AHO, including a round face, short stature, short third and fourth fingers and subcutaneous calcifications. The patient also exhibited a fixed right ankle joint, plantar flexion deformity, and right heel deformity, as well as right ankle and popliteal fossa hyperextension and a tendon contracture that prevented the right heel from touching the ground during walking. Results from the nervous system and cardiovascular exams were unremarkable. As shown in Table 1, laboratory findings showed extremely low corrected serum calcium concentrations at 1.03 mmol/L (2.1–2.7 mmol/L), serum phosphorus concentrations of 2.45 mmol/L (0.83–1.48 mmol/L), and serum magnesium concentrations of 0.76 mmol/L (0.70–1.15 mmol/L), PTH of 249.40 pg/mL (12–65 pg/mL), serum vitamin D level of 17.74 ng/mL (30–100 ng/mL), thyrotropin (TSH) of 7.408 μIU/mL (0.35–4.94 μIU/mL), FT4 of 10.17 pmol/L (9.01–19.05 pmol/L), alkaline phosphatase of 351.3 U/L (34–121 U/L), white blood cell count of 2.4 × 10^9^/µL (4–10 × 10^9^/µL), and an elevated serum creatine kinase of 921.7 U/L (22–269 U/L). The electrocardiogram (ECG) in this case showed sinus rhythm along with a prolonged corrected QT interval of 482 ms, which is a sign of hypocalcemia. The left-hand X-ray revealed brachydactyly of the third and fourth fingers, which is a common feature of AHO. Computed tomography (CT) of the right lower limb showed ectopic calcification extending from the gastrocnemius space of the upper segment of the right leg to the rear of the knee joint, indicating POH. Multiple calcifications were visible on the head CT scan in the basal ganglia, soft tissues underneath the scalp, and both frontal lobes on either side, which is a common manifestation of AHO. Clinical signs of AHO included the patient’s round face, short neck, brachydactyly of the third and fourth digits, subcutaneous calcification, low calcium, and increased PTH. The patient also had a history of congenital hypothyroidism. Laboratory analysis revealed the clinical PTH levels, indicating a diagnosis of PHP. Genetic testing confirmed that the patient had PHP 1a due to a GNAS gene intron5 splicing mutation chr20:57478848 C.432+2T>CP.? (NM_000516.7 (Figure 5a). The patient’s mother had the same genetic mutation (Figure 5b) but did not exhibit AHO clinical signs or laboratory abnormalities, with a height of 140 cm and a weight of 37.2 kg, as well as normal levels of calcium, phosphorus, TSH, T3, T4, PTH, and sex hormone. His father’s genetic test was normal (Figure 5c). Calcium and calcitriol supplementation improved the patient’s symptoms. Following treatment, the patient’s seizures abated and he remained asymptomatic during his hospital stay. His adjusted calcium level was 1.83 mmol/L, which was within the normal range, but his PTH level remained high at 189 pg/mL at follow-up 1 week after discharge. As his subcutaneous calcification did not improve, surgery was performed. Postoperative pathology confirmed extensive ectopic ossification lesions in the soft tissues of the lower leg and fibrous adipose tissue with ossification (Figure 6).

**Table 1:** The results of laboratory tests of the patient, compared to the normal reference range

Laboratory results	Patient values	Normal reference range
White blood cell count(10^3^/µL)	2.4	4–10
Hemoglobin (g/dL)	11.6	12–15
Platelet count (10^3^/µL)	273	150–400
Urea (mmol/L)	3.5	2.76–8.07
Creatinine (µmol/L)	48	53–97
Sodium (mmol/L)	141.1	135–145
Potassium (mmol/L)	3.96	3.5–5.3
Chloride (mmol/L)	98.8	95–105
Glucose (mmol/L)	4.4	3.3–5.5
C-reactive protein (mg/L)	<5	0–5
Vitamin D (ng/mL)	17.74 low	30–100
Albumin (g/L)	47	35–50
Corrected calcium(mmol/L)	1.20 low	2.1–2.7
Phosphorus (mmol/L)	2.45 high	0.83–1.48
Magnesium (mmol/L）	0.76	0.70–1.15
Alkaline phosphatase (U/L)	351.3	34–121
Creatine kinase (U/L)	921.7	22–269
Myoglobin (ng/mL)	48	25–58
Parathyroid hormone (pg/mL)	249.40 high	12–65
Free triiodothyronine (nmol/L)	3.17	2.63–5.70
Free thyroxine (nmol/L)	10.17	9.01–19.05
Thyroid-stimulating hormone (μIU/mL)	7.408 high	0.350–4.940

**Informed consent:** Informed consent has been obtained from all individuals included in this study.
**Ethical approval:** The research related to human use has been complied with all the relevant national regulations, institutional policies, and in accordance with the tenets of the Helsinki Declaration and has been approved by the Ethics Committee of Quzhou People’s Hospital according to the Helsinki Declaration.

## 2 Discussion and conclusions

The PTH signaling pathway deficiency in PHP is classified into three types: PHP 1, PHP 2, and pseudopseudohypoparathyroidism (PPHP). PHP 1 is further subdivided into three distinct subtypes: 1a, 1b, and 1c. PHP 1a is caused by maternal GNAS exons 1–13 mutations that result in characteristic abnormalities known as AHO and are associated with resistance to G protein-coupled receptors (GPCRs) [5]. PPHP, on the other hand, is caused by paternal GNAS mutations in exons that also cause most of these AHO features, but without evidence of hormonal resistance (Table 2).

**Table 2:** Clinical and biochemical characteristics of PHP 1a and PPHP

Consequences of mutations	PHP 1a	PPHP
GNAS mutations	Maternal	Paternal
Resistance at GPCRs	Yes	Rare cases with mild hormonal resistance
Serum calcium and phosphorus	Hypocalcemia and hyperphosphatemia	Normal
AHO	Yes	Yes
PHO	Rare	Yes
Birth weights	Close to average	Low

Legend: GPCRs (G protein-coupled receptors), AHO (Albright hereditary osteodystrophy), POH (progressive osseous heteroplasia).

The patient in this case exhibited resistance to both thyroid hormone and PTH, was born with congenital hypothyroidism, and now has PTH resistance. Ectopic calcifications are common in patients with AHO, particularly in the subcutaneous tissue and brain parenchyma, with the most common manifestation of calcification in the basal ganglia of the brain. However, it remains unclear whether intracranial calcification is the underlying cause of seizures in PHP patients. Recent studies [6] suggest that intracranial calcification is not a reliable etiological cause of epilepsy in PHP patients; dysfunction of GPCR signaling pathways and a reduction of serum calcium may underlie the etiology of seizures in PHP. The molecular cause of GNAS-associated HO is still unclear, but recent studies have shown that GNAS inactivation can regulate the differentiation and function of not only osteoblasts but also osteoclasts and osteocytes [7,8]. GNAS inactivation leads to heterotopic bone formation within subcutaneous tissue by changing the connective tissue microenvironment, thereby promoting osteogenic differentiation of tissue-resident mesenchymal progenitors. Both the sonic hedgehog and hippo signaling pathways contribute to HO formation [7,8]. Most AHO-related heterotopic bone develops through intramembranous bone formation and is limited to the dermis and subcutaneous tissue. However, in rare cases with Gsα mutations, ossification progresses to the deep connective tissue and skeletal muscle, a condition known as POH. Both PHP 1a and POH are caused by heterozygous inactivating mutations in the GNAS gene, with POH-like progressive and prominent ectopic calcification mostly caused by paternal GNAS mutations. In this case, CT of the patient’s lower extremity showed ectopic calcification and ossification in the tissue, suggesting POH, which significantly restricted joint movement and led to the development of a fixed flexion deformity of the patient’s right ankle joint. Differential diagnoses were also made for the patient’s joint fixation, mainly including chronic arthritis, post-traumatic injury, pseudoarthrosis, and soft tissue calcification. Currently, there has been no effective treatment for HO, but nonsteroidal anti-inflammatory drugs and local irradiation may be helpful in preventing HO, whereas surgical therapy to excise existing HO is limited by a high frequency of recurrence and complications. Surgical resection of the HO may be recommended for patients with severe or lamellar heterotopic calcifications in deep tissue or high-pressure loaded joints, and bisphosphonates can be utilized to limit postoperative recurrence of HO because they have the ability to effectively suppress bone turnover. This patient developed intracranial calcification and deep tissue ossification, resulting in the joint fixation and flexion deformity. His HO and joint fixation deformity were treated by surgery, and the pathological results of the postoperative tissue confirmed the patient’s HO. Inhibition of Yes-associated protein-Sonic hedgehog may be a promising therapeutic strategy for treating HO [7].

There are only a few case reports of POH overlap syndrome with PHP 1a [9,10,11,12,13] (Table 3), and the mechanism underlying this overlap syndrome remains unclear. Genetic variation, epigenetic modifications, and environmental factors may contribute to POH overlap syndrome. Several GNAS mutations have been reported to lead to POH overlap syndrome with PHP 1a, including the donor splice site in intron 5 C.432+2T>G of GNAS, which was first identified in a patient with iPPSD2 [14]. However, little is known about how this splice site mutation can lead to iPPSD2. The case presented in this report showed a heterozygous C.432+2T>C(P.?) variant in the boy and his mother, suggesting maternal inheritance of the GNAS mutation. As the rarity of POH/PHP 1a, further studies of the GNAS heterozygous mutation C.432+2T>C may reveal the factors involved in POH overlap syndrome.

**Table 3:** Clinical and molecular characteristics of cases of PHP 1a with POH-like phenotype reported in the literature

Pt ID	Gender	Age of onset/diagnosis	GNAS mutation position	AHO	Ectopic calcification	Hormone resistance patterns	Familial transmission/mutated allele
1 [9]	F	6 months/7 years	no	Yes	Arms, abdomen, legs, and right foot	rPTH/rTSH	De novo/maternal
2 [10]	M	4 months/16 months	c.546del exon7	Yes	Lower limbs, buttocks, and back	rPTH/rTSH	De novo/maternal
3 [11]	M	Postnatal/1 year	c.85 > T	Yes	Cervical, truncular, arms	TSH( ND) rPTH	De novo/maternal
4 [12]	F	6 months/3 years	c.568_571del exon7	Yes	Heterotopic ossification	rPTH/rTSH	Inherited/maternal
5 [13]	M	Postnatal/13 years 6 months	p.D189MfsTer14	No	Left floor and right hand	rPTH/rTSH	De novo/maternal

Legend: M (male), F (female), rPTH (parathyroid hormone resistance), rTSH (thyroid-stimulating hormone resistance), AHO (Albright hereditary osteodystrophy), ND (not determined), de novo (no parental inheritance).

In conclusion, we presented a rare case of POH overlap syndrome with a maternal GNAS mutation, congenital hypothyroidism, tonic-clonic seizures, AHO features, deep ectopic ossification, and joint fixation deformity. Further studies of POH and PHP 1a cases will help us to have a better understanding of POH overlap syndrome.

## References

[1] Jüppner H. Pseudohypoparathyroidism: complex disease variants with unfortunate names. J Mol Endocrinol. 2023;72(1):e230104. 10.1530/JME-23-0104 PMC10843601 37965945

[2] Takatani R, Kubota T, Minagawa M, Inoue D, Fukumoto S, Ozono K, et al. Prevalence of pseudohypoparathyroidism and nonsurgical hypoparathyroidism in Japan in 2017: A Nationwide Survey. J Epidemiol. 2023;33(11):569–73. 10.2188/jea.JE20220152 PMC10518380 36123043

[3] Del Sindaco G, Berkenou J, Pagnano A, Rothenbuhler A, Arosio M, Mantovani G, et al. Neonatal and early infancy features of patients with inactivating PTH/PTHrP signaling disorders/pseudohypoparathyroidism. J Clin Endocrinol Metab. 2023;108(11):2961–9. 10.1210/clinem/dgad236 PMC10583975 37098127

[4] Elli FM, Mantovani G. Pseudohypoparathyroidism, acrodysostosis, progressive osseous heteroplasia: different names for the same spectrum of diseases? Endocrine. 2021;72(3):611–8. 10.1007/s12020-020-02533-9 PMC8159830 33179219

[5] Jüppner H. Molecular definition of pseudohypoparathyroidism variants. J Clin Endocrinol Metab. 2021;106(6):1541–52. 10.1210/clinem/dgab060 PMC8118362 33529330

[6] Qi Z, Li Z, Gao Q, Dong L, Lin J, Peng K, et al. Characterizing cerebral imaging and electroclinical features of five pseudohypoparathyroidism cases presenting with epileptic seizures. Behav Neurol. 2022;2022:8710989. 10.1155/2022/8710989 PMC9391127 35992960

[7] Cong Q, Liu Y, Zhou T, Zhou Y, Xu R, Cheng C, et al. A self-amplifying loop of YAP and SHH drives formation and expansion of heterotopic ossification. Sci Transl Med. 2021;13(599):eabb2233. 10.1126/scitranslmed.abb2233 PMC8638088 34162750

[8] McMullan P, Germain-Lee EL. Aberrant bone regulation in albright hereditary osteodystrophy dueto gnas inactivation: mechanisms and translational implications. Curr Osteoporos Rep. 2022;20(1):78–89. 10.1007/s11914-022-00719-w 35226254

[9] Eddy MC, Jan de Beur SM, Yandow SM, McAlister WH, Shore EM, Kaplan FS, et al. Deficiency of the alpha-subunit of the stimulatory G protein and severe extraskeletal ossification. J Bone Miner Res. 2000;15(11):2074–83. 10.1359/jbmr.2000.15.11.2074 11092390

[10] Gelfand IM, Hub RS, Shore EM, Kaplan FS, Dimeglio LA. Progressive osseous heteroplasia-like heterotopic ossification in a male infant with pseudohypoparathyroidism type Ia: a case report. Bone. 2007;40(5):1425–8. 10.1016/j.bone.2006.12.058 17321228

[11] Lebrun M, Richard N, Abeguilé G, David A, Coëslier Dieux A, Journel H, et al. Progressive osseous heteroplasia: a model for the imprinting effects of GNAS inactivating mutations in humans. J Clin Endocrinol Metab. 2010;95(6):3028–38. 10.1210/jc.2009-1451 20427508

[12] Elli FM, deSanctis L, Ceoloni B, Barbieri AM, Bordogna P, Beck-Peccoz P, et al. Pseudohypoparathyroidism type Ia and pseudo-pseudohypoparathyroidism: the growing spectrum of GNAS inactivating mutations. Hum Mutat. 2013 Mar;34(3):411–6. 10.1002/humu.22265 23281139

[13] Ozaki K, Mituboshi A, Nagai M, Nishiyama A, Nishimura G, Morisada N, et al. Mild progressive osseous heteroplasia overlap syndrome with PTH and TSH resistance appearing during adolescence and not early childhood. Endocrine. 2021 Dec;74(3):685–9. 10.1007/s12020-021-02821-y 34254228

[14] Le Norcy E, Reggio-Paquet C, de Kerdanet M, Mignot B, Rothenbuhler A, Chaussain C, et al. Dental and craniofacial features associated with GNAS loss of function mutations. Eur J Orthod. 2020;42(5):525–33. 10.1093/ejo/cjz084 31696922

